# Grain filling of early-season rice cultivars grown under mechanical transplanting

**DOI:** 10.1371/journal.pone.0224935

**Published:** 2019-11-07

**Authors:** Jiana Chen, Fangbo Cao, Shuanglü Shan, Xiaohong Yin, Min Huang, Yingbin Zou

**Affiliations:** 1 Crop and Environment Research Center, College of Agronomy, Hunan Agricultural University, Changsha, China; 2 Guangxi Key Laboratory of Rice Genetics and Breeding, Rice Research Institute, Guangxi Academy of Agricultural Sciences, Nanning, China; Shandong University, CHINA

## Abstract

High yields of mechanized intensive rice-based cropping systems, e.g. double-season cropping using early- and late-season rice, are important to ensure national food security in China. However, few studies addressing the relationship between grain weight and grain yield of early-season rice under machine-transplanted conditions. A field experiment was conducted to determine the critical grain-filling characteristics and related physiological aspects that contribute to high grain weight in machine-transplanted early-season rice. The results showed that grain yield was significantly positively correlated with grain weight but not with panicles per m^2^, spikelets per panicle, and spikelet-filling percentage. Furthermore, this study demonstrated that there was a significant positive correlation between grain weight and mean grain-filling rate, which was significantly positively correlated with harvest index and grain cytokinin content. These results indicate that high grain-filling rate driven by good transport of assimilates to grains and strong grain sink strength is responsible for high grain weight in machine-transplanted early-season rice.

## Introduction

Rice is the staple food for more than 65% of the population in China. In order to ensure national food security, intensive rice-based cropping systems have been extensively developed in China [1]. Double-season cropping using early- and late-season rice is a major intensive rice-based system in China [2]. However, in recent years, urban expansion has led to a labor shortage and an increase in wages for agricultural production in China [3], which have been major problems for double-season rice production in China [4]. The planting area of double-season rice has sharply decreased from 19.3 million ha in 1985 to 11.8 million ha in 2016 in China [5]. To overcome this situation, several simplified cultivation technologies have been developed for rice production in China [6] and large-scale farming has been used to utilize labor effectively. In recent years, farmland rental has increased in China under government guidance, and a new class of farmers who obtain farmland on lease for large-scale farming has emerged [7, 8]. The development of large-scale farming has promoted the adoption of machine-transplanting technologies for rice production in China [9].

Grain yield of rice is determined by number of panicles per unit land area, number of spikelets per panicle, spikelet-filling percentage, and grain weight [10]. However, there have been contradictory findings on the primary determinant of grain yield in rice. Zhang et al. [11] compared grain yield and yield components among super hybrid, ordinary hybrid, and inbred rice cultivars. Their results showed that higher grain yield was attributable to more spikelets per panicle in super hybrid rice than in ordinary hybrid and inbred rice. Huang et al. [12] calculated the contributions of yield components to grain yield in eight super hybrid rice cultivars and found that number of panicles per m^2^ generally had the highest positive contribution to grain yield. Hongthong et al. [13] compared grain yield between two super hybrid cultivars differing in grain weight, and the results indicated that developing rice cultivars with high grain weight is a possible approach to achieve high grain yield.

These contradictory findings are generally obtained from single-season rice, while little information is available on double-season rice, especially early-season rice. In 2017, we conducted a preliminary field experiment to investigate the relationships between grain yield and yield components in early-season rice grown under mechanical transplanting (S1 File). The results showed that grain yield was closely related to grain weight but not to the other three yield components (S1 Fig). Obviously, increasing grain weight is a feasible way to improve grain yield of machine-transplanted early-season rice and highlights the need for greater fundamental understanding of the physiological processes governing grain weight in early-season rice under machine-transplanted conditions.

Grain weight is the product of the duration and rate of grain filling. Jones et al. [14] analyzed the relationships between grain weight with the duration and rate of grain filling in fifteen rice cultivars and found that grain weight was positively and significantly related to grain-filling rate. Huang and Zou [15] also found that high grain-filling rate was responsible for the high grain weight. Furthermore, grain filling is closely related to source-sink traits in rice. Sikder and Gupta [16] reported that source capacity (i.e. assimilate supply) was the main determinant of grain filling in rice. However, Yang et al. [17] observed that poor grain filling of *japonica*/*indica* hybrid rice was not attributable to source limitation but to poor transport of assimilates to grains. In addition, grain filling is reported to be controlled by grain sink strength (e.g. endosperm cell number) [18], which is closely related to hormones (e.g. cytokinins and abscisic acid) in the grain [19]. An increase in grain cytokinins can increase the number of endosperm cells and alter the intensity and direction of assimilate flow, while an increase in grain abscisic acid can enhance the remobilization of pre-stored assimilates to the grains [19].

In the present study, grain-filling characteristics and source-sink traits were compared among six early-season rice cultivars grown under mechanical transplanting. The objective of this study was to determine the critical grain-filling characteristics and related physiological aspects that contribute to the high grain weight in machine-transplanted early-season rice.

## Materials and methods

### Ethics statements

No specific permissions were required for the activities conducted in this study. The field used in this study is neither privately owned nor protected. The experiments did not involve endangered or protected species.

### Site and soil

**Fig 1:**
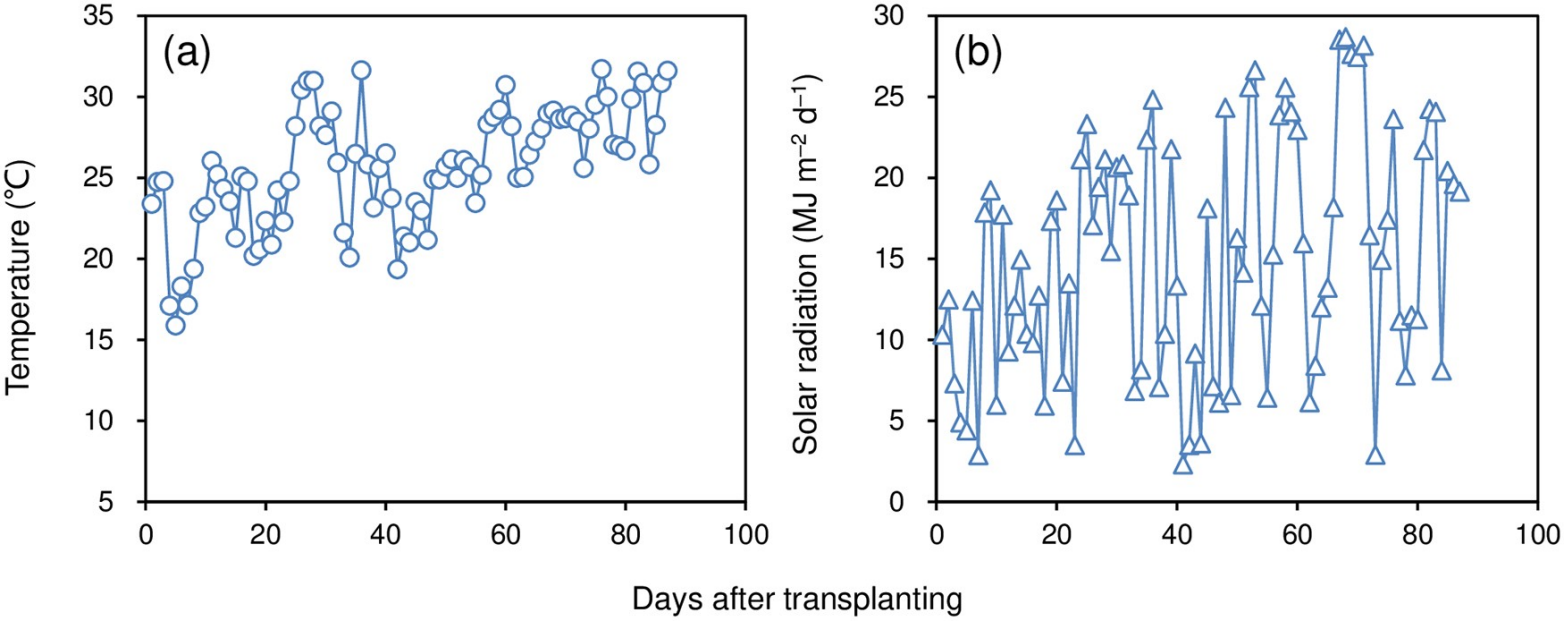
Daily mean temperature (a) and solar radiation (b) in the early rice-growing season in 2018.

A field experiment was conducted in Yongan Town (28°09′ N, 113°37′ E, 43 m asl), Hunan Province, China in the early rice-growing season in 2018. The average daily temperature and solar radiation during the season were 25.6 °C and 15.0 MJ m^–2^ d^–1^, respectively (Fig 1). The soil of the experimental field was a clay with the following properties: pH 6.25, 37.3 g organic matter kg^–1^, 172 mg available N kg^–1^, 18.2 mg available P kg^–1^, and 80.7 mg available K kg^–1^. The soil test was based on samples taken from the 0–20 cm layer before the experiment was begun.

### Cultivars and management

Six rice cultivars, including Luliangyou 996 (Lu 18S × 996), Xiangzaoxian 24 (Xiangzaoxian 11 × Xiangzaoxian 7), Xiangzaoxian 32 (Xiangzaoxian 11 × Xiangzaoxian 17), Xiangzaoxian 42 (Feng 9 × Fengyouzao 11), Zhuliangyou 189 (Zhu 1S × R189), and Zhuliangyou 819 (Zhu 1S × Hua 819), were used in the experiment. These cultivars were selected because (1) they represented a diversity of agronomic characteristics in the preliminary experiment and (2) they have been widely grown by rice farmers in the study region. The cultivars were arranged in a randomized complete-block design with three replicates and a plot size of 40 m^2^.

Seeds were sown according to the procedures described by Huang et al. [20]. Seedlings were raised in mat nursery, and 25-day-old seedlings were transplanted on 20 April with a high-speed rice transplanter (PZ80–25, Dongfeng Iseki Agricultural Machinery Co., Ltd., Xiangyang, China). Transplanting was done at a spacing of 25 cm × 11 cm. Missing plants were replanted by hand at 7 days after transplanting to ensure a uniform plant population. Urea (Hubei Saning Chemical Co., Ltd., Zhijiang, China) was used as N fertilizer and applied in three splits: 67.5 kg N ha^–1^ (50%) as basal fertilizer (1 day before transplanting), 27.0 kg N ha^–1^ (20%) at early-tillering (7 days after transplanting), and 40.5 kg N ha^–1^ (30%) at panicle initiation. Superphosphate (Guangdong Zhanhua Co., Ltd., Zhanhua, China) was used as phosphorus (67.5 kg P_2_O_5_ ha^–1^) and applied as basal fertilizer. Potassium chloride (Sinochem Group, Beijing, China) was used as potassium (135 kg K_2_O ha^–1^) and split equally as basal fertilizer and at panicle initiation. The experimental field was kept flooded from transplanting until 7 days before maturity. Insects, diseases, and weeds were intensively controlled by chemicals to avoid yield loss. Validamycin was used to control rice sheath blight and false smut. Triazophos, buprofezin, and phoxim were used to control rice borer, planthopper, and leaf roller, respectively. Weeds were controlled by herbicide penoxsulam and hand-pulling. The major pests and diseases stresses that caused yield reductions were not observed in the experiment field due to the intensive crop management practices.

### Sampling and measurements

About 150 main-stem panicles that headed on the same day were tagged in each plot. Seven tagged panicles were sampled randomly from each plot, starting at 3 days after full heading (when more than 80% of plants showed panicles) at a 3-day interval until maturity. The grains in the middle part of the sampled panicle were hand threshed, hulled, and oven-dried at 70 °C for about 72 h to a constant weight. The grain-filling process (i.e. changes in grain weight with days after full heading) was fitted using the logistic equation, and grain-filling parameters including initial grain-filling rate (GR_0_), maximum grain-filling rate (GR_max_), mean grain-filling rate (GR_mean_), and active grain-filling duration (GD_active_) were calculated according to Shi et al. [21]. In addition, three tagged panicles were sampled from each plot at 6, 9, and 12 days after full heading. The grains in the middle part of the sampled panicle were hand threshed and blended, frozen in liquid nitrogen for 1 min, and stored at –80 °C until assayed. Cytokinins and abscisic acid in hulled grains were extracted and purified following the procedure of Li et al. [22] and then analyzed using a high-performance liquid chromatography system (Agilent 1100 Series, Agilent Technologies Inc., Palo Alto, CA, USA) with an Agilent TC C-18 column (250 mm × 4.6 mm, 5 μm) at 30 °C and a mixture of water (600 ml), acetic acid (6 ml), and methyl alcohol (400 ml) as the mobile phase at a flow rate of 0.8 ml min^–1^.

Twelve hills were sampled from each plot at full heading and maturity. The plants sampled at full heading were separated into stems, leaves, and panicles and oven-dried at 70 °C to a constant weight. Pre-heading biomass production was the total dry matter of stems, leaves, and panicles. The plants sampled at maturity were hand threshed after counting panicle number. Filled spikelets were separated from unfilled spikelets by submerging them in tap water. The number of filled spikelets was counted using an automatic seed counter (SLY-A, Zhejiang Top Instrument Co., Ltd., Hangzhou, China), and the number of unfilled spikelets was manually counted. Dry weights of straw and filled and unfilled spikelets were determined after oven drying at 70 °C to a constant weight. Total biomass production was the summation of dry matter of straw and filled and unfilled spikelets. Post-heading biomass production was the difference between total and pre-heading biomass production. Translocation of pre-heading biomass to spikelets was the difference between filled spikelet weight and post-heading biomass production [23].

Yield components including panicles per m^2^, spikelets per panicle, spikelet-filling percentage, and grain weight as well as source-sink parameters including pre-heading biomass production per spikelet (BP_pre_, pre-heading biomass production/filled spikelet number), post-heading biomass production per spikelet (BP_post_, post-heading biomass production/filled spikelet number), total biomass production per spikelet (BP_total_, total biomass production/filled spikelet number), translocation of pre-heading biomass to each spikelet (TB_pre_, translocation of pre-heading biomass to spikelets/filled spikelet number), and harvest index (HI, filled spikelet weight/total biomass production) were calculated. Grain yield was determined from a 5 m^2^ area in each plot and adjusted to the standard moisture content of 14%.

### Statistical analysis

Data were subjected to one-way analysis of variance after Shapiro-Wilk normality test (Statistix 8, Analytical software, Tallahassee, FL, USA). Means of cultivars were compared based on the least significant difference test (LSD) at the 0.05 probability level. The correlations between grain yield, yield components, grain-filling characteristics, and source-sink traits were evaluated using Pearson’s correlation analysis, and the significance was set at the 0.05 probability level.

## Results

### Grain yield and yield components

Zhuliangyou 189 and Zhuliangyou 819 produced grain yields of more than 7.5 t ha^–1^, which was approximately 8% higher than that in Luliangyou 996 (*P* < 0.05), 16% higher than that in Xiangzaoxian 42 (*P* < 0.05), 25% higher than that in Xiangzaoxian 24 (*P* < 0.05), and 34% higher than that in Xiangzaoxian 32 (*P* < 0.05) (Table 1). Zhuliangyou 189 had the highest panicles per m^2^, which was 10% higher than that in Zhuliangyou 819 (*P* < 0.05), 23% higher than that in Xianzaoxian 42, and 42% higher than that in Luliangyou 996 (*P* < 0.05). Luliangyou 996 had the highest spikelets per panicle, which was 16–18% higher than those in Xiangzaoxian 42 and Zhuliangyou 819 (*P* < 0.05), and 36–47% higher than those in Zhuliangyou 189 (*P* < 0.05). Xiangzaoxian 32, and Xiangzaoxian 24. Xiangzaoxian 32 had the highest spikelet-filling percentage, but it was not significantly higher than those in Xiangzaoxian 24, Xiangzaoxian 42, Zhuliangyou 189, and Zhuliangyou 819 (*P* > 0.05). Luliangyou 996 and Zhuliangyou 819 had a grain weight of more than 30 mg, which was about 8% higher than that in Zhuliangyou 819 (*P* < 0.05), 13% higher than that in Xiangzaoxian 42 (*P* < 0.05), and 21% higher than those in Xiangzaoxian 24 and Xianzaoxian 32 (*P* < 0.05).

**Table 1:** Grain yield and yield components of six early-season rice cultivars.

Cultivar	Grain yield (t ha^–1^)	Panicles m^–2^	Spikelets panicle^–1^	Spikelet filling (%)	Grain weight (mg)
Luliangyou 996	6.99 ± 0.14 b	273 ± 8 d	122 ± 8 a	78.0 ± 3.9 b	31.0 ± 0.2 a
Xiangzaoxian 24	6.04 ± 0.08 cd	375 ± 4 ab	83 ± 1 c	84.5 ± 1.2 ab	25.5 ± 0.1 d
Xiangzaoxian 32	5.63 ± 0.24 d	361 ± 6 ab	86 ± 2 c	86.1 ± 1.3 a	25.3 ± 0.2 d
Xiangzaoxian 42	6.50 ± 0.34 bc	317 ± 12 c	105 ± 5 b	80.9 ± 1.5 ab	27.2 ± 0.2 c
Zhuliangyou 189	7.62 ± 0.10 a	389 ± 22 a	90 ± 2 c	82.3 ± 3.0 ab	30.6 ± 0.2 a
Zhuliangyou 819	7.51 ± 0.26 a	353 ± 19 b	103 ± 3 b	80.4 ± 3.5 ab	28.6 ± 0.1 b

Within a column, means not sharing any letter are significantly different by the LSD test at the 0.05 probability level.

### Grain-filling characteristics

**Fig 2:**
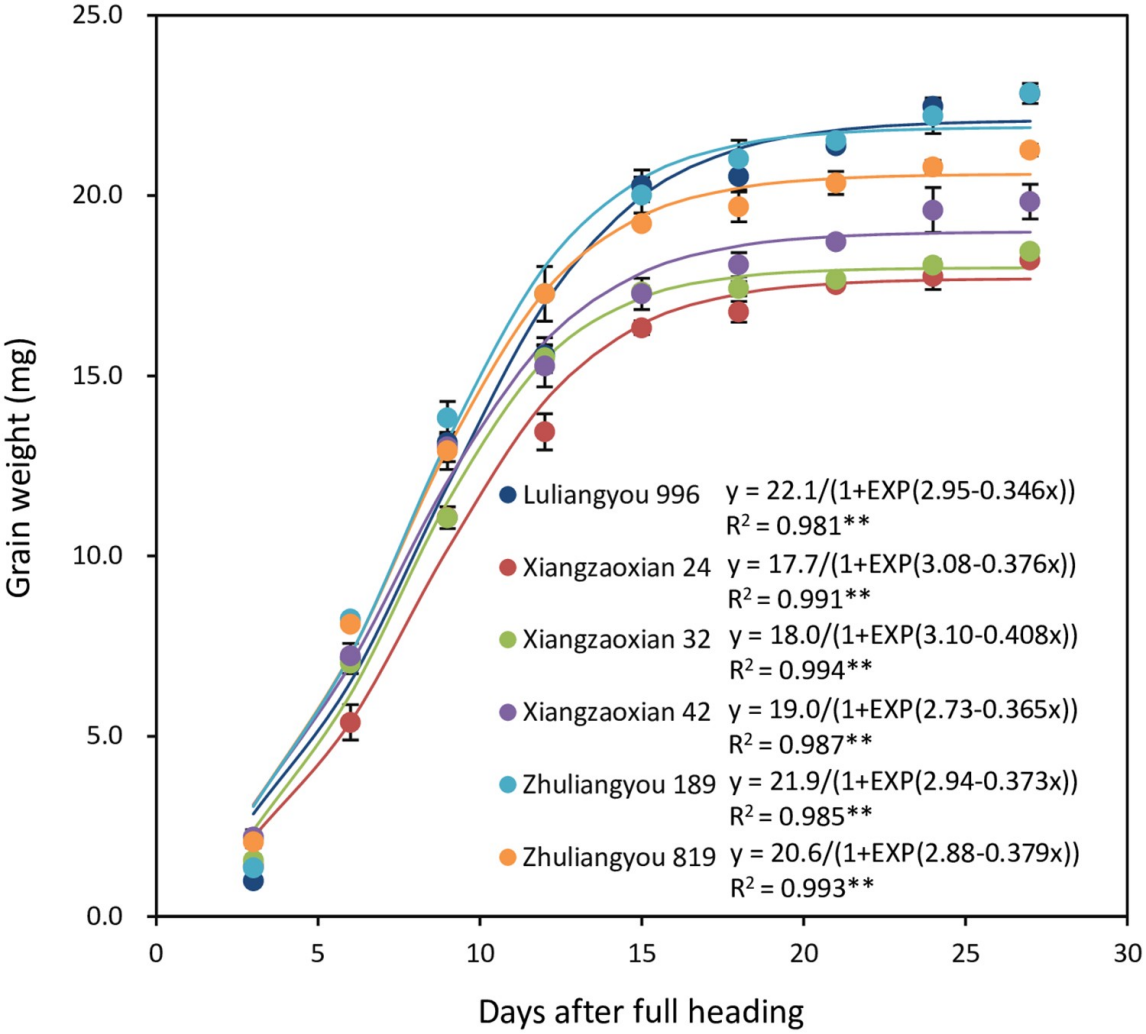
Grain-filling process fitted by logistic equation for six early-season rice cultivars. ** denotes significance at the 0.01 probability level.

The grain-filling process was well fitted by the logistic equation (R^2^ = 0.981–0.994, *P* < 0.01) for all six cultivars (Fig 2). Xiangzaoxian 42 had the highest GR_0_, which was 3% higher than that in Zhuliangyou 189 and Zhuliangyou 819, 11% higher than that in Luliangyou 996, 33% higher than that in Xiangzaoxian 32, and 43% higher than that in Xiangzaoxian 24 (Table 2). GR_max_ was highest in Zhuliangyou 189, followed by Zhuliangyou 819, Luliangyou 996, Xiangzaoxian 32, Xiangzaoxian 42, and Xiangzaoxian 24. The highest GR_mean_ was recorded in Zhuliangyou 189, which was 4% higher than that in Zhuliangyou 819, 7% higher than that in Luliangyou 996, 14% higher than that in Xiangzaoxian 32 and Xiangzaoxian 42, and 24% higher than that in Xiangzaoxian 24. Luliangyou 996 had the longest GD_active_, followed by Xiangzaoxian 24, Zhuliangyou 189, Xiangzaoxian 42, Zhuliangyou 819, and Xiangzaoxian 32.

**Table 2:** Initial grain-filling rate (GR_0_), maximum grain-filling rate (GR_max_), mean grain-filling rate (GR_mean_), and active grain-filling duration (GD_active_) of six early-season rice cultivars.

Cultivar	GR_0_ (mg grain^–1^ d^–1^)	GR_max_ (mg grain^–1^ d^–1^)	GR_mean_ (mg grain^–1^ d^–1^)	GD_active_ (d)
Luliangyou 996	0.36	1.91	1.01	17.0
Xiangzaoxian 24	0.28	1.66	0.87	16.0
Xiangzaoxian 32	0.30	1.84	0.95	14.8
Xiangzaoxian 42	0.40	1.73	0.95	15.5
Zhuliangyou 189	0.39	2.04	1.08	15.8
Zhuliangyou 819	0.39	1.95	1.04	15.4

### Source-sink traits

Luliangyou 996 had the highest BP_pre_, which was 20% higher than that in Xiangzaoxian 24 (*P* < 0.05), 31% higher than those in Xiangzaoxian 42 and Zhuliangyou 189 (*P* < 0.05), 42% higher than that in Zhuliangyou 819 (*P* < 0.05), and 48% higher than that in Xiangzaoxian 24 (*P* < 0.05) (Table 3). Zhuliangyou 189 had the highest BP_post_, and although it was not significantly higher than those in Zhuliangyou 819 and Xiangzaoxian 42 (*P* > 0.05), it was 28% higher than that in Xiangzaoxian 32 (*P* < 0.05), and 44–50% higher than those in Xiangzaoxian 24 and Luliangyou 996 (*P* < 0.05). Luliangyou 996 and Zhuliangyou 189 had a similar BP_total_, with an average of 48.3 mg, which was 7% higher than that in Xiangzaoxian 42 (*P* < 0.05), 10% higher than that in Zhuliangyou 189 (*P* < 0.05), 11% higher than that in Xiangzaoxian 24 (*P* < 0.05), and 21% higher than that in Xiangzaoxian 32 (*P* < 0.05). TB_pre_ was highest in Luliangyou 996, which was 73–167% higher than those in other five cultivars (*P* < 0.05). Zhuliangyou 819 had the highest HI, which was not significantly higher than those in Luliangyou 996 and Zhuliangyou 189 (*P* > 0.05), but it was 4% higher than that in Xiangzaoxian 32 (*P* < 0.05), 7% higher than that in Xiangzaoxian 42 (*P* < 0.05), and 12% higher than that in Xiangzaoxian 24 (*P* < 0.05).

**Table 3:** Pre-heading biomass production per spikelet (BP_pre_), post-heading biomass production per spikelet (BP_post_), total biomass production per spikelet (BP_total_), translocation of pre-heading biomass to each spikelet (TB_pre_), and harvest index (HI) of six early-season rice cultivars.

Cultivar	BP_pre_ (mg)	BP_post_ (mg)	BP_total_ (mg)	TB_pre_ (mg)	HI (%)
Luliangyou 996	34.4 ± 0.9 a	14.3 ± 0.8 c	48.7 ± 0.1 a	12.3 ±1.0 a	0.55 ± 0.00 ab
Xiangzaoxian 24	28.7 ± 1.0 b	14.9 ± 1.2 c	43.6 ± 0.4 c	7.1 ± 1.1 b	0.50 ± 0.01 d
Xiangzaoxian 32	23.2 ± 1.1 d	16.8 ± 0.9 bc	40.0 ± 0.3 d	5.0 ± 0.8 b	0.54 ± 0.01 b
Xiangzaoxian 42	26.3 ± 0.7 bc	18.7 ± 0.4 ab	45.0 ± 0.4 b	4.6 ± 0.5 b	0.52 ± 0.00 c
Zhuliangyou 189	26.3 ± 0.5 bc	21.5 ± 0.9 a	47.8 ± 0.7 a	4.8 ± 0.8 b	0.55 ± 0.01 ab
Zhuliangyou 819	24.3 ± 0.8 cd	19.6 ± 0.7 ab	43.9 ± 0.4 bc	5.0 ± 0.7 b	0.56 ± 0.00 a

Within a column, means not sharing any letter are significantly different by the LSD test at the 0.05 probability level.

**Fig 3:**
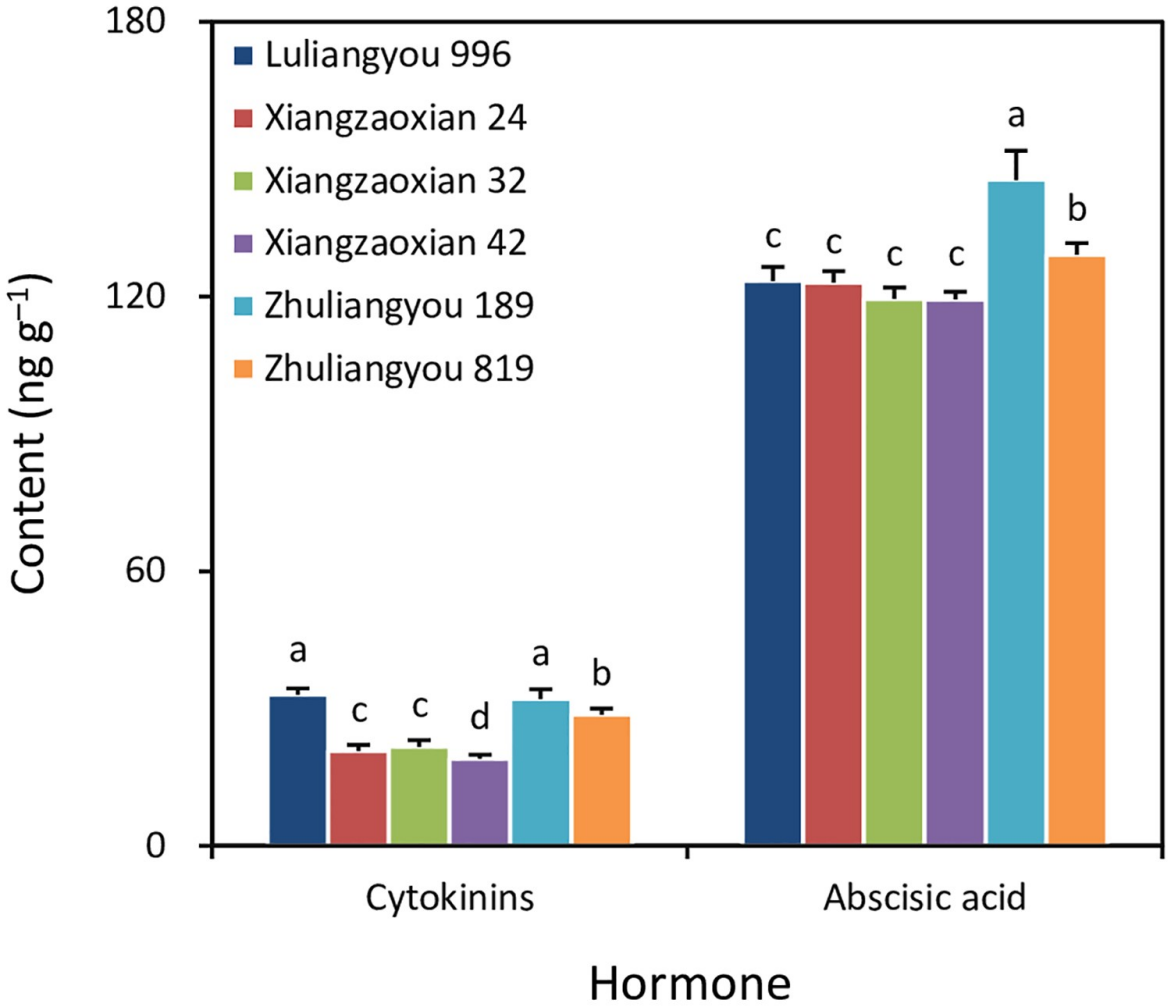
Grain cytokinin and abscisic acid contents during the rapid grain-filling period of six early-season rice cultivars. Each column is the mean of three replicates. Error bars show standard errors. Means not sharing any letter are significantly different by the LSD test at the 0.05 probability level.

Luliangyou 996 and Zhuliangyou 189 had a similar grain cytokinin content, showing an average of 32.5 ng g^–1^, which was 13% higher than that in Zhuliangyou 819 (*P* < 0.05), 51–57% higher than those in Xiangzaoxian 24 and Xiangzaoxian 32 (*P* < 0.05), and 71% higher than that in Xiangzaoxian 42 (*P* < 0.05) (Fig 3). Zhuliangyou 189 had the highest grain abscisic acid content, which was 13% higher than that in Zhuliangyou 819 (*P* < 0.05), and 18–22% higher than those in Luliangyou 996, Xiangzaoxian 24, Xiangzaoxian 32, and Xiangzaoxian 42 (*P* < 0.05).

### Correlations between grain yield and related traits

**Fig 4:**
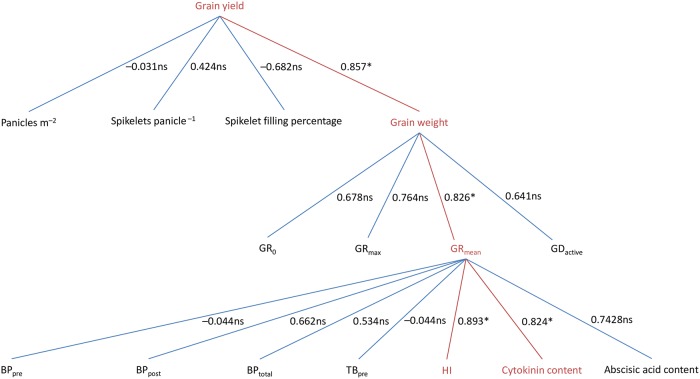
Pearson’s correlation coefficients between grain yield, yield components, grain-filling characteristics, and source-sink traits in early-season rice. The data used for analysis are presented in Tables 1–3 and Fig 3. ns and * denote non-significant and significant, respectively, at the 0.05 probability level (*n* = 6). GR_0_, initial grain-filling rate; GR_max_, maximum grain-filling rate; GR_mean_, mean grain-filling rate; GD_active_, active grain-filling duration; BP_pre_, pre-heading biomass production per spikelet; BP_post_, post-heading biomass production per spikelet; BP_total_, total biomass production per spikelet; TB_pre_, translocation of pre-heading biomass to each spikelet; HI, harvest index.

Grain yield was significantly positively correlated with grain weight (*P* < 0.05), but not with panicles per m^2^, spikelets per panicle, or spikelet-filling percentage (*P* > 0.05) (Fig 4). In addition, grain weight was significantly and positively correlated with GR_mean_ (*P* < 0.05) but not with GR_0_, GR_max_, and GD_active_ (*P* > 0.05). Furthermore, GR_mean_ was positively correlated with HI and grain cytokinin content (*P* < 0.05) but not with BP_pre_, BP_post_, BP_total_, TB_pre_, or grain abscisic acid content (*P* > 0.05).

## Discussion

Various agronomic characteristics were observed among the six tested early-season rice cultivars. The results of the present study indicate that grain weight is the critical yield component contributing to high grain yield in early-season rice. This finding is consistent with the results of our preliminary study (S1 Fig), which showed that grain yield was closely related to grain weight but not to the other three yield components, i.e., panicles per m^2^, spikelets per panicle, and spikelet-filling percentage. However, these results are not in agreement with those observed in single-season rice by Zhang et al. [11] and Huang et al. [12], who reported that higher grain yield was attributed to higher panicle number or larger panicle size (spikelets per panicle). This difference might be because early- and single-season rice have different vegetative growth durations and climatic conditions during the vegetative growth period, which can affect the development and growth of tillers and panicles. In general, early-season rice has a shorter vegetative growth duration and lower temperature during the vegetative growth period than single-season rice [24]. Therefore, it might be more difficult to form either more panicles or larger panicles in early-season rice compared to single-season rice.

Although grain weight is associated with both the rate and duration of grain filling, it has been reported that grain weight is mainly determined by grain-filling rate [14, 15]. The results of our present study, namely that grain weight was significantly positively correlated with GR_mean_, also support this view. This result could also be partly explained by the climatic conditions, especially the temperature during the grain-filling period. It has been well documented that high temperature during the grain-filling period can induce early plant senescence, reduce photosynthetic capacity, and shorten grain-filling duration; however, it can increase the remobilization of nonstructural carbohydrate from the vegetative tissues to the grain and accelerate grain-filling rate [21, 25, 26]. In the early rice-growing season, high temperature always occurs during the grain-filling period [24, 25]. Hence, increasing grain-filling rate should be more feasible than prolonging the grain-filling duration to increase grain weight for early-season rice.

There have been contradictory reports on the relationship between grain filling and source-sink traits in rice [16, 17]. In summary, it is not known whether grain filling is determined by source capacity or transport of assimilates to grains. In the present study, no significant correlations were observed between GR_mean_ and source capacity parameters including BP_pre_, BP_post_, BP_total_, and TB_pre_, while a significant positive relationship was found between GR_mean_ and HI. These results indicate that good transport of assimilates to the grain was partially responsible for the high grain-filling rate of early-season rice.

In addition, the high grain-filling rate of the early-season rice could also be partly attributed to strong grain sink strength in this study, where a significant positive correlation was found between GR_mean_ and grain cytokinin content during the rapid grain-filling period. This finding is consistent with that reported by Yang and Zhang [19]. However, there is also a different result between the present study and the report of Yang and Zhang [19], namely that a significant relationship between GR_mean_ and grain abscisic acid content during the rapid grain-filling period was reported by Yang and Zhang [19] but was not observed in this study. This difference might be related to the experimental conditions. The experiments reported in Yang and Zhang [19] were mostly conducted under different water management practices including soil drying, while the experiment in the present study was done only under flooding conditions. It has been well documented that water management practices have a major influence on grain abscisic acid content in rice [27]. In general, soil drying during the grain-filling period can lead to an increase in grain abscisic acid content.

## Conclusions

Selecting cultivars with high grain weight is a feasible way to achieve high grain yield in machine-transplanted early-season rice. The high grain weight is attributed to a high grain-filling rate, which is driven by good transport of assimilates to grains and strong grain sink strength.

## Supporting information

S1 File General details of preliminary field experiment. (PDF)

S1 Fig Relationships between grain yield and yield components in early-season rice. Each point is the mean of three replicates for one cultivar. Error bars show standard errors.
(TIF)
