# Supplementary material for: Grain filling of early-season rice cultivars grown under mechanical transplanting
Source: PLoS One. 2019 Nov 7;14(11):e0224935. doi: 10.1371/journal.pone.0224935 (PMC6837445; doi:10.1371/journal.pone.0224935)
Supplement: S1 File — (PDF) [file pone.0224935.s001.pdf]

Methods. General details of preliminary field experiment.

A field experiment was conducted in Santang Town (26°53′ N, 112°28′ E, 71 m asl), Hunan Province, China in the early rice-growing season in 2017. The soil of the experimental field was a clay with the following properties: pH = 5.86, organic matter = 31.0 g kg^{-1}, available N = 145 mg kg^{-1}, available P = 14.1 mg kg^{-1}, and available K = 187 mg kg^{-1}. The soil test was based on samples taken from the 0–20 cm layer before the experiment was begun.

Ten rice cultivars were arranged in a randomized complete-block design with three replicates and a plot size of 15 m^2. Twenty five-day-old seedlings were transplanted on 24 April with a high-speed rice transplanter [2ZGQ-8B(NSD8), Kubota Agricultural Machinery (Suzhou) Co., Ltd., Suzhou, China]. Transplanting was done at a spacing of 25 cm × 12 cm. The missing plants were replanted by hand at 7 days after transplanting to ensure a uniform plant population. Nitrogen was applied in three splits: 75 kg N ha^{-1} as basal fertilizer (1 day before transplanting), 45 kg N ha^{-1} at early-tillering (7 days after transplanting), and 30 kg N ha^{-1} at panicle initiation. Phosphorus (75 kg P_2O_5 ha^{-1}) was applied as basal fertilizer. Potassium (150 kg K_2O ha^{-1}) was split equally at basal fertilization and panicle initiation. The experimental field was kept flooded from transplanting until 7 days before maturity. Insects, diseases, and weeds were intensively controlled by chemicals to avoid yield loss.

Ten hills were sampled from each plot at maturity. The sampled plants were hand threshed after counting the number of panicles. Filled spikelets were separated from unfilled spikelets by submerging them in tap water. The number of filled spikelets was counted using an automatic seed counter (SLY-A, Zhejiang Top Instrument Co., Ltd., Hangzhou, China), and the number of unfilled spikelets was manually counted. Dry weight of filled spikelets was determined after oven drying at 70 °C to a constant weight. Yield components including panicles per m^2, spikelets per panicle, spikelet-filling percentage, and grain weight were calculated. Grain yield was determined from a 5 m^2 area in each plot and adjusted to the

standard moisture content of 14%.

The relationships between grain yield and yield components were evaluated using linear regression analysis (Statistix 8, Analytical software, Tallahassee, FL, USA).

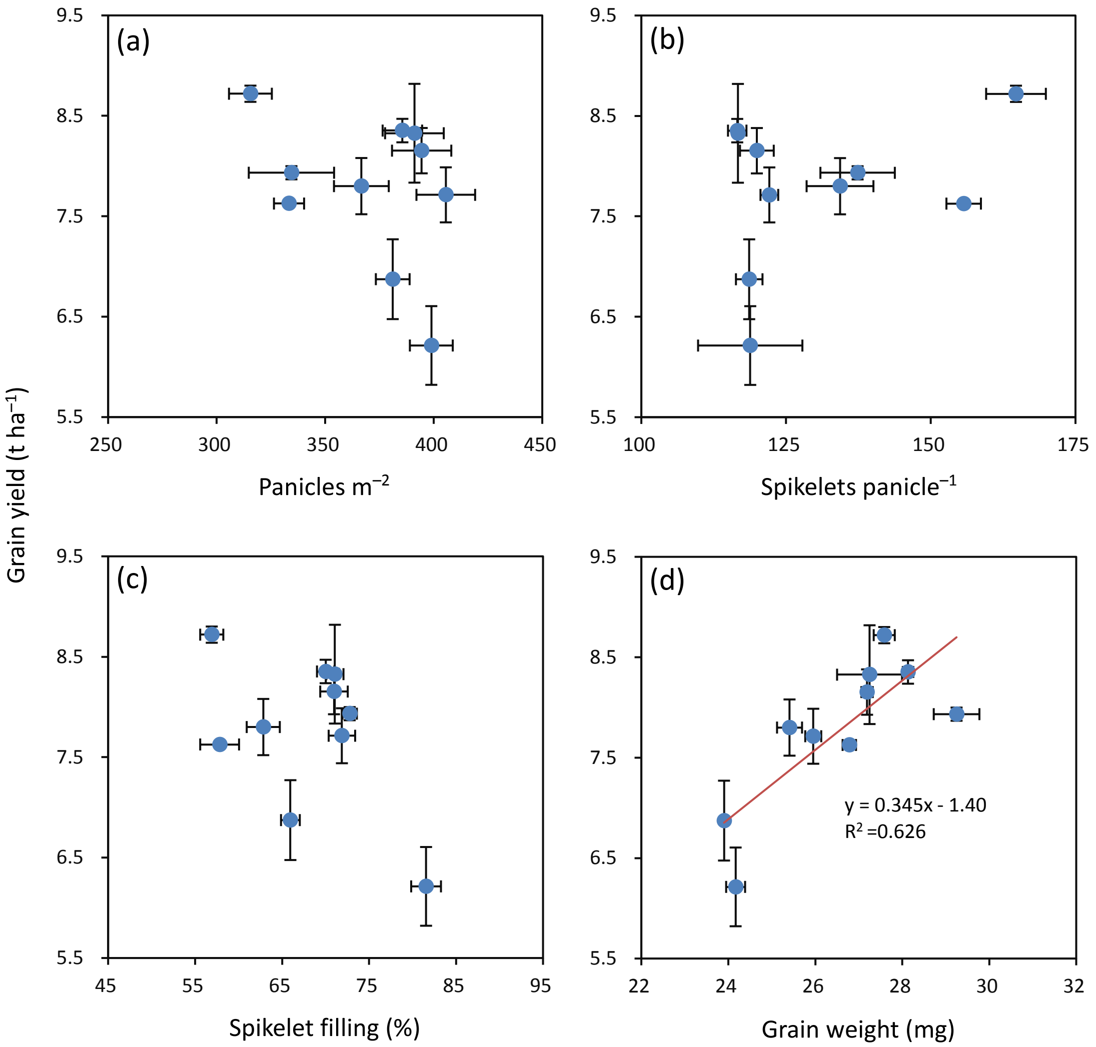

Figure S1. Relationships between grain yield and yield components in early-season rice. Each point is the mean of three replicates for one cultivar. Error bars show standard errors.
