# Supplementary figures and images for: Grain filling of early-season rice cultivars grown under mechanical transplanting
Source: PLoS One. 2019 Nov 7;14(11):e0224935. doi: 10.1371/journal.pone.0224935 (PMC6837445; doi:10.1371/journal.pone.0224935)

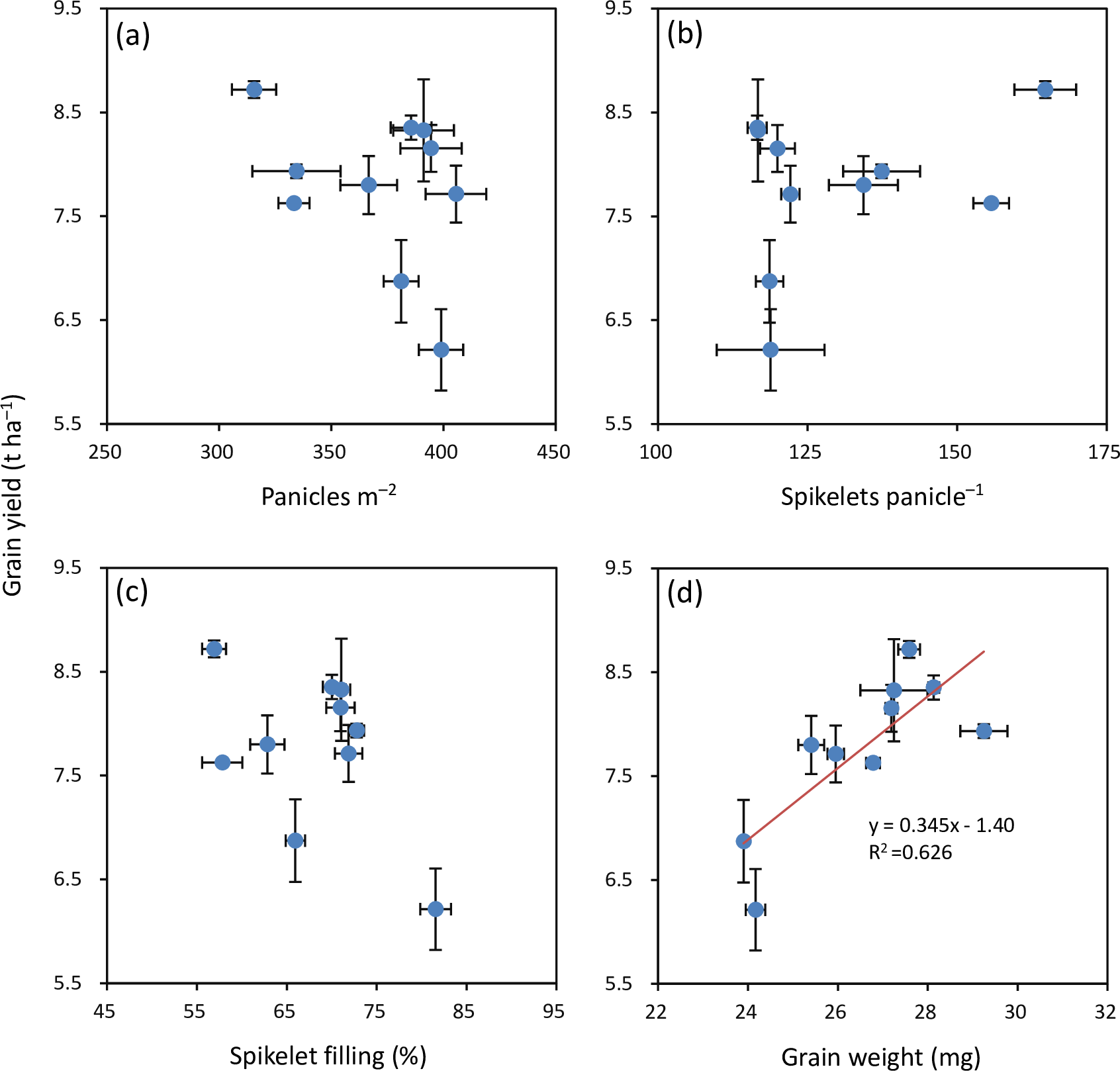

Supplement: S1 Fig — Each point is the mean of three replicates for one cultivar. Error bars show standard errors. (TIF) [file pone.0224935.s002.tif]
